# Recent Advances in Targetable Therapeutics in Metastatic Non-Squamous NSCLC

**DOI:** 10.3389/fonc.2016.00112

**Published:** 2016-05-04

**Authors:** Pranshu Bansal, Diaa Osman, Gregory N. Gan, George R. Simon, Yanis Boumber

**Affiliations:** ^1^Department of Internal Medicine, Division of Hematology/Oncology, University of New Mexico Comprehensive Cancer Center, University of New Mexico School of Medicine, Albuquerque, NM, USA; ^2^Hematology/Oncology Fellowship Program, University of New Mexico Comprehensive Cancer Center, University of New Mexico School of Medicine, Albuquerque, NM, USA; ^3^Section of Radiation Oncology, University of New Mexico Comprehensive Cancer Center, Albuquerque, NM, USA; ^4^Department of Thoracic and Head/Neck Medical Oncology, Division of Cancer Medicine, University of Texas MD Anderson Cancer Center, Houston, TX, USA; ^5^Cancer Genetics, Epigenetics, and Genomics Research Program, University of New Mexico Comprehensive Cancer Center, Albuquerque, NM, USA

**Keywords:** NSCLC, EGFR, ALK, ROS1, HER2, BRAF, c-MET, VEGFR2

## Abstract

Lung adenocarcinoma is the most common subtype of non-small cell lung cancer (NSCLC). With the discovery of epidermal growth factor receptor (*EGFR*) mutations, anaplastic lymphoma kinase (*ALK*) rearrangements, and effective targeted therapies, therapeutic options are expanding for patients with lung adenocarcinoma. Here, we review novel therapies in non-squamous NSCLC, which are directed against oncogenic targets, including EGFR, ALK, ROS1, BRAF, MET, human epidermal growth factor receptor 2 (HER2), vascular endothelial growth factor receptor 2 (VEGFR2), RET, and NTRK. With the rapidly evolving molecular testing and development of new targeted agents, our ability to further personalize therapy in non-squamous NSCLC is rapidly expanding.

## Introduction

In recent years, advances in lung cancer are occurring at an accelerated pace. With several new targeted therapies approved last year for non-squamous non-small cell lung cancer (NSCLC) alone, it is now one of the most active areas in oncology. Approvals include one vascular endothelial growth factor receptor 2 (VEGFR2) inhibitor, two EGFR-targeting drugs, one anaplastic lymphoma kinase (ALK)-targeting drug, and one ROS1 inhibitor. This review summarizes breakthroughs that have already happened, and some promising ongoing areas of investigation where new drug approvals are anticipated.

## Epidermal Growth Factor Receptor

Identification of oncogenic epidermal growth factor receptor (EGFR) mutations in NSCLC has ushered a new era of targeted therapies in metastatic NSCLC. These mutations lead to activation of EGFR, now effectively targeted by an ever-increasing list of tyrosine kinase inhibitors (TKIs). Exon 19 deletion and exon 21 L858R substitutions account for 90% of all EGFR + NSCLC (1). Activating EGFR mutation occur in ~10% Caucasians (2), higher in never smokers (30%) vs. ex-smokers (5%) and current smokers (3%) (2, 3).

Erlotinib and gefitinib are FDA-approved, first-generation reversible inhibitors of both wild-type and mutant EGFR. These are accepted first-line therapies in patients with known EGFR-sensitizing mutations in metastatic NSCLC and have shown overall response rate (ORR) up to 75% and advantage in progression-free survival (PFS) over chemotherapy in several clinical studies (4–6). Class side effects with these agents are primarily skin rash and diarrhea. Despite high ORR and improved PFS, long-term impact of such therapies is limited by the development of resistance.

Afatinib, a second-generation irreversible inhibitor of EGFR and human epidermal growth factor receptor 2 (HER2), also FDA-approved, is used as a frontline therapy in metastatic NSCLC patients with EGFR-sensitizing mutations. Side-effect profile is similar to the first-generation drugs. Two phase III trials, LUX-Lung 3 and LUX-Lung 6, have shown improved PFS and ORR with first-line afatinib compared to chemotherapy (6, 7). Recent subgroup analysis from LUX-Lung 3 and LUX-Lung 6 trials showed significantly improved OS in patients with exon 19 deletion treated with afatinib compared to chemotherapy: 33.3 vs. 21.1 months and 31.4 vs. 18.4 months (8). This OS difference was not seen in patients harboring L858R mutation (8). LUX-lung 7 is a phase II trial comparing afatinib with gefitinib in advanced NSCLC. Preliminary analysis showed that patients treated with afatinib had 27% reduction in risk of progression compared to patients treated with gefitinib. The benefits were seen in both L858R and exon 19 deletion subgroups (9).

The mechanisms of acquired resistance to first-generation EGFR inhibitors can be divided into three groups: secondary mutations, bypass signaling, and phenotypic alterations. The most common resistance mechanism seen in ~50% cases is the development of genetic alterations of EGFR, commonly a secondary mutation like T790M, a threonine to methionine substitution in exon 20. This mutation leads to an enhanced affinity for ATP and steric hindrance, reducing the ability of ATP-competitive, reversible EGFR TKIs to bind to the tyrosine kinase domain of EGFR (10–14). The second mechanism is the activation of additional signaling pathways, including MET, HER2, and CRKL amplification, AXL overexpression, and KRAS and BRAF mutations (15, 16). The third mechanism is histologic transformation into SCLC (17). In ~30% cases, resistance mechanisms are unknown.

One potential way to overcome T790M mutation is the use irreversible EGFR inhibitor afatinib. However, clinical studies with afatinib in patients progressing on the first-generation TKIs have shown a modest ORR of ~10% and PFS of ~4 months (18). Afatinib in combination with cetuximab showed an improved ORR of 30% in T790M+ patients after progression on first-generation TKIs; however, toxicity was a significant concern with this combination (19).

Rociletinib and osimertinib are third-generation TKIs with specificity for EGFR T790M over wild-type EGFR. Osimertinib showed response rate of 60% in phase I/II studies in T790M+ tumors and ~30% in tumors negative for T790M in previously treated EGFR + NSCLC (20). This is a significant advancement over chemotherapy or afatinib/cetuximab combination, which leads to FDA approval of the drug for EGFR T790M+ patients progressing on first/second-generation TKIs (21–24). Rociletinib is another EGFR T790M selective TKI, awaiting FDA approval. Initial rociletinib phase I/II data reported an ORR of 60% and disease control rate (DCR) of 90%; however, initial data contained immature findings, including unconfirmed responses, and the revised ORR was significantly lower at 28–34% (25, 26). Both drugs have a lower incidence of grade 3/4 rash and diarrhea. The most common toxicity with rociletinib is hyperglycemia secondary to IGF1 inhibition (25). ASP8273 and HM61713 are third-generation EGFR TKIs currently in development (NCT02500927, NCT02485652, and NCT02588261).

As with the first- and second-generation EGFR TKIs, tumor evolution leads to resistance to third-generation TKI’s. So far, the data for resistance to third-generation EGFR TKI’s is mostly preclinical. Niederst et al. showed additional C797S EGFR mutations in EGFR T790M cell lines made resistant *in vitro* to third-generation TKIs. They showed resistant cell lines, harboring C797S and T790M mutations in cis-allelic conformation, were resistant to all types of TKIs; however, if these two mutations were expressed in trans conformation, the cells became resistant to third-generation TKIs but retained sensitivity to first-generation TKIs. Similarly, EGFR C797S mutant but T790M wild-type cell lines retained sensitivity to first-generation TKIs (27). In a separate study of 12 T790M+ patients who underwent tumor biopsy post-progression on rociletinib, 6 patients became T790M wild-type, 2 T790M wild-type cancers underwent SCLC transformation, 3 T790M-positive cancers acquired *EGFR* amplification, and 1 patient had T790 wild-type and mutant cell populations coexisting in the tumor (28). Other proposed mechanisms of third-generation TKI resistance include epithelial mesenchymal transition (EMT) (21), activation of the MAPK kinase pathway (22), and IGF1R bypass signaling (23).

Combination of an EGFR TKI with VEGF inhibitor, such as bevacizumab, has been studied in patients with EGFR-sensitizing mutations. In a phase II trial comparing erlotinib (E) to erlotinib plus bevacizumab (E + B), median PFS was 16 months compared to 9 months in the E arm (24). E + B combination has shown activity in T790M+ NSCLC, and a recent study showed a median PFS of 16 months in T790M+ NSCLC compared to 10.5 months in T790M wild-type patients (29).

MET amplification occurs in ~3–7% of untreated patients and ~21% patients previously treated with EGFR TKIs (30, 31). Tivantinib is a MET TKI, which was studied in combination with erlotinib compared to erlotinib alone. Combination showed improvement in PFS without advantage in OS; subgroup analysis showed a trend for OS advantage in high MET expression subgroup (32). INC280, another MET TKI, has shown promise in the initial dose escalation and combination phase I study with first-generation EGFR TKI, ongoing in patients who have progressed on EGFR TKI monotherapy (NCT01610336).

## Anaplastic Lymphoma Kinase

The EML4–ALK fusion oncogene arises from an inversion on the short arm of chromosome 2 [Inv (2) (p21p23)] that joins exons 1–13 of EML4 to exons 20–29 of ALK (33). The EML4 fusion partner mediates ligand-independent dimerization and/or oligomerization of ALK, resulting in constitutive kinase activity. *ALK*+ NSCLC represents ~4–5% of NSCLC patients and is much higher among never/light smokers, up to 22% (34, 35).

Crizotinib is a small-molecule that competes with ATP tyrosine kinase activity. Initially developed as a c-MET inhibitor, it was later found to have potent inhibitory activity against ALK and ROS1 (36, 37). In phase I/II studies, crizotinib demonstrated ORR in ~60% of patients with *ALK*-positive NSCLC and PFS of 7–10 months (38–40). In a recent phase III study, crizotinib was compared to chemotherapy in untreated patients with ALK+ NSCLC, demonstrating a significant improvement in ORR and PFS. No significant OS benefit was shown since 70% of patients crossed over to crizotinib arm after progression on chemotherapy (41). Crizotinib is an FDA-approved, first-line therapy in ALK+ NSCLC metastatic patients.

In spite of the dramatic initial responses, patients develop resistance to crizotinib after a median of 8.9–10.5 months (42). In particular, central nervous system (CNS) is a common site of relapse in patients with ALK+ metastatic NSCLC (43). In a study of 28 patients treated with crizotinib, 13 developed CNS relapses (44). Nevertheless, recent update from the crizotinib phase III trial showed a delay in the onset of CNS relapse compared to chemotherapy (41).

Crizotinib resistance mechanisms can be ALK-dependent or -independent. ALK-dependent resistance includes ALK amplification/copy number gain and additional genetic alterations, including secondary mutations of the ALK kinase domain that preserve ALK signaling (45, 46). Of these, a secondary mutation L1196M, which interferes with binding of crizotinib, is the most common (47). This is very similar to T790M mutation in EGFR+ NSCLC. Additional resistance mutations that occur less frequently include G1269A, C1156Y, L1152R, G1202R, S1206Y, 1151Tins, F1174C, and D1203N.

The ALK-independent mechanisms of resistance involve activation of bypass signaling pathways, such as the EGFR, HSP90, MET, KRAS, or KIT. Analysis of 16 NSCLC patients treated with crizotinib and re-biopsied at progression showed ALK amplification in 13%, ALK mutations in 31%, and KRAS or EGFR pathway activation in 31%; 19% patients had HSP90, KIT, or HER2 activation (46).

In 2014, FDA approved ceritinib, a second-generation ALK inhibitor, when impressive results from a single-arm study of 163 patients with ALK+ positive metastatic NSCLC showed median ORR of 44%. Majority (91%) patients had progressed on prior crizoitinb. This trial also included patients with baseline brain metastasis (60%) (47). Two phase III trials comparing ceritinib to chemotherapy in treatment naïve and previously treated patients are ongoing (NCT01828099, NCT01828112).

Alectinib is another FDA-approved, second-generation ALK inhibitor with activity in crizotinib-resistant ALK+ metastatic NSCLC. A phase II study with 138 patients progressing on crizotinib had 50% ORR and DCR of 79% (48). In a frontline, phase I/II study of crizotinib-naive patients with ALK+ NSCLC treated with alectinib showed an impressive ORR of 93.5% (49). Alectinib has shown activity in patients with CNS metastasis; one study showed an intracranial ORR of 52% in patients progressing on crizotinib (50). A phase III clinical trial comparing alectinib and crizotinib upfront was stopped early as it met its primary endpoint (ALEX trial, NCT02075840).

Mechanisms of resistance to ceritinib and alectinib are undergoing active investigation. G1202R ALK mutation is pan-resistant to crizotinib, ceritinib, and alectinib (43, 51). Multiple ALK and ROS1 inhibitors are in development. Lorlatinib (PF-06463922) is an ALK/ROS1 novel CNS-penetrant inhibitor with preclinical activity against the G1202R mutation that demonstrated encouraging activity in resistant patients: 40% PRs, including patients progressing following crizotinib ± ceritinib; intracranial responses were also observed (52, 53). Brigatinib (AP26113) is an exciting TKI with dual activity against EGFR T790M and ALK L1196M mutations. The results from phase I/II showed significant activity in ALK+ patients. Of 72 evaluable pts, 72% responded: 45/65 (69%) with prior crizotinib and all 7 crizotinib-naive pts. Median duration of response (DOR) was 49 weeks (54).

## ROS1

The ROS1 oncogene encodes an orphan receptor tyrosine kinase related to ALK. ROS1 rearrangements occur in ~1–2% of patients with NSCLC, mainly non-smokers or light smokers (55). Phase 1 expansion trial with crizotinib in ROS1 rearrangement-positive NSCLC patients demonstrated marked antitumor activity with 72% ORR and 17.6 months median DOR, which led to its FDA approval in 2016 (56). Similar to experience with EGFR and ALK, crizotinib resistance develops with acquired mutations, such as G2032R and L2155S, in ROS1 kinase domain (57, 58). Preclinical studies with lorlatinib look encouraging, as it has shown activity against novel mutations in both ALK and ROS1, which cause resistance to first- and second-generation TKIs (52). In phase I/II trial, 22 patients with ALK+ or ROS1+ NSCLC received lorlatinib, which was well tolerated with encouraging clinical activity (59). Phase II part of this trial is ongoing (NCT01970865).

## BRAF

Oncogenic BRAF mutation is found in ~3–4% of NSCLC, usually non-overlapping with other oncogenic driver mutations with ~50% cases harboring the characteristic V600E mutation (60, 61). Unlike EGFR and ALK, BRAF mutations commonly occur in smokers (60–62). Vemurafenib and dabrafenib are BRAF-targeting TKIs, approved in BRAF-mutated metastatic melanoma. They have shown promise in early-phase trials in NSCLC. A basket phase II trial looking into the efficacy of vemurafenib in different tumors harboring BRAF mutations included 20 patients with advanced NSCLC. The ORR was 42%, superior to other cancers types (63). Dabrafenib showed a ORR of 32% in 78 patients with BRAF-mutated NSCLC; 2-stage phase II study with the second phase evaluating the combination of MEK inhibitor trametinib and dabrafenib is ongoing. Interim analysis showed an encouraging ORR of 63% in 24 patients (64).

## c-MET Inhibitors

The MET amplification is oncogenic in 3–7% of NSCLC and confers resistance to EGFR in ~21% cases (31, 32). In addition to amplification, MET gene alterations, namely exon 14 internal deletions and mutations, are oncogenic in a small fraction of NSCLC patients (65). MET receptor tyrosine kinase and its ligand hepatocyte growth factor (HGF) are implicated in tumor cell proliferation, invasion, and angiogenesis in NSCLC (66). Responses with anti-MET TKIs, such as crizotinib and cabozantinib, in MET mutant and amplified NSCLC have been reported (67, 68).

Glesatinib (MGCD265, Mirati) is an oral TKI targeting MET and AXL. A phase I/II trial has been initiated in MET mutant or MET-amplified NSCLC. Early data from phase Ib part had shown confirmed PRs and significant tumor reductions; phase II part is ongoing (NCT02544633).

INC280 is a highly selective small-molecule MET inhibitor with preclinical activity in human tumor models; early-phase trial is initiated of INC280 alone or in combination with chemotherapy or erlotinib (NCT02468661).

## Vascular Endothelial Growth Factor Receptor 2

Blockade of VEGFR2 signaling inhibits formation, proliferation, and migration of new blood vessels (69). Ramucirumab is a human IgG1 monoclonal antibody that targets the extracellular domain of VEGFR2, FDA-approved in metasatic NSCLC, in combination with docetaxel, after a phase III trial demonstrated OS benefit over docetaxel alone (69).

Cabozantinib is a VEGFR2/MET inhibitor assessed in a phase II trials in pretreated patients with NSCLC (*n* = 125). Cabozantinib alone or in combination with erlotinib significantly improved PFS over erlotinib in pts with EGFR wild-type NSCLC (70).

## Human Epidermal Growth Factor Receptor 2

Human epidermal growth factor receptor 2 (ERBB2) belongs to ErbB receptor tyrosine kinase family along with EGFR (HER1), HER3, and HER4. Ligand binding and subsequent homo and heterodimerization of these receptors activate EGFR, HER3, and HER4. In this regard, HER2 is unique, as no HER2 ligand has been identified. HER2 is the preferred binding partner of ERBB receptors, in particular EGFR. HER2/EGFR heterodimers have an increased potential for signaling than EGFR homodimers (71). HER2 overexpression (~35%) and amplification (~20%) have been reported in NSCLC (72–74). Clinical results with HER2-directed therapy in these patients have not been convincing (75–77). HER2 mutations, including exon 20 insertions, are oncogenic in breast and lung cancer (77–81). HER2 mutations occur in ~2–5% of NSCLC, more commonly in Asian, non-smoker, and female populations (80, 81). HER2-targeted therapies in HER2-mutant population are an ongoing area of research. The largest retrospective study looking at HER2-targeted agents in this population demonstrated 50% ORR among 16 HER2-mutant patients, primarily treated with trastuzumab and afatinib (82). A trial (NCT02369484) initiated with afatinib in HER2 mutation-positive NSCLC is ongoing.

Neratinib is another irreversible pan-HER inhibitor, which has shown activity in trastuzumab-resistant breast cancer (83). It was evaluated in a phase I study in combination with temsirolimus (mTOR inhibitor) and showed activity in 2/6 HER2-mutant NSCLC (84). Based on these results, a phase II study of neratinib with and without temsirolimus is ongoing (NCT01827267).

Dacomitinib, an irreversible pan-EGFR inhibitor, demonstrated ORR of 13% in HER2-mutant NSCLC patients in a phase II study (85).

## RET

The *RET* gene encodes a RET family receptor tyrosine kinase. Activating somatic point mutations in RET occur in medullary thyroid cancer (86). Recurrent translocations between RET and various fusion partners occur in ~12% NSCLC (87–89). The prevalence is higher among non-smokers, negative for other driver mutations (90).

Cabozantinib showed encouraging results in a phase II study of 16 patients with RET fusion-positive disease, 7/16 had PR with median PFS of 7 months and OS 10 months (91). Studies in RET+ NSCLC with lenvatinib (NCT01877083), apatinib (NCT02540824), vandetinib (NCT01823068), and ponatinib (NCT01813734) are ongoing.

## NTRK

NTRK gene encodes for tropomyosin receptor kinase (Trk) protein. Vaishnavi et al. in their pioneering work have shown *MPRIP*–*NTrk1* and *CD74*–*NTrk1* fusions leading to constitutive TrkA activity in 3.3% patients with NSCLC (92). In the same study, authors reported oncogenic TPM3–NTRK1 fusion that has also been reported in a small fraction of colon cancer (93). Stransky et al. reported TRIM24–NTRK2 gene fusion in a NSCLC patient (94).

Entrectinib (RXDX-101) is a highly potent inhibitor of TRK as well as ROS1 and ALK. In a phase I trial, entrectinib has demonstrated clinical activity in TRK-fusion-positive advanced solid malignancies. Trials with this drug and other novel NTRK inhibitors are ongoing [(95), NCT02576431, NCT01639508].

## Conclusion

Advances in targeted therapy for metastatic non-squamous NSCLC have now expanded from EGFR and ALK to additional oncogenic targets, including ROS1, BRAF, RET, HER2, NTRK, and MET. Testing for these genes is now standard in many centers and is recommended by the NCCN (96). Identification of new drivers leading to effective personalized therapy is an exciting but challenging task in today’s world. As we know, most targetable mutations are rare, and therefore development of standardized therapies calls for innovative ways to improve our clinical and translational knowledge. In basket trials, patients are included based on molecular aberration regardless of histology, whereas umbrella trials include patients of single tumor types (97). The latter involves a group of two or more enrichment designs, or sub-studies, connected through a central infrastructure overseeing screening and identification of patients and centralized tissue analysis for standardized genotyping (98).

The other major issue we face with molecularly targeted agents is the inevitable emergence of resistance. We are now venturing into the era of resistance to third-generation TKI’s in EGFR/ALK therapies, and the treatment paradigm changes with every successive generation of inhibitors. Patients progressing on first-generation EGFR inhibitors are expected to undergo tumor re-biopsies; and tumor heterogeneity and false-negative results make future treatments more challenging.

**Figure 1:**
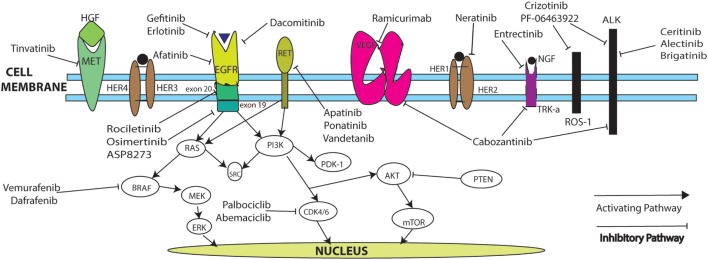
**Targeted pathways for NSCLC**.

Table 1 summarizes ongoing trials in NSCLC, and Figure 1 describes mechanisms of action. Novel drug testing and development of rational drug combination in frontline and recurrent settings in NSCLC remains one of the most exciting, rapidly evolving areas in oncology, with hopes to dramatically increase the numbers of long-term survivors with stage IV disease.

**Table 1:** **Selective ongoing non-squamous NSCLC targeted therapy trials**.

Drug class and target	Investigational agent	Comparator arm	Trial ID number	Phase
EGFR inhibitors	ASP8273	–	NCT02500927	II
	HM61713 (BI 1482694)	–	NCT02485652	II
	Gefitinib + INC280	–	NCT01610336	Ib/II
	Osimertinib + navitoclax	–	NCT02520778	Ib
	Erlotinib + bevacizumab (BELIEF trial)	–	NCT01562028	II
ALK inhibitors	PF-06463922 (ALK/ROS1 inhibitor)	–	NCT01970865	I/II
	AP26113	–	NCT01449461	I/II
	TSR-011	–	NCT02048488	I/IIa
	RXDX-101	–	NCT02097810	I/IIa
	X-396	–	NCT01625234	I/II
	Alectinib (ALEX study)	Crizotinib	NCT02075840	III
EGFR or ALK inhibitor + combination checkpoint inhibitor	Ipilimumab/nivolumab plus erlotinib or crizotinib	–	NCT01998126	IB
BRAF/MEK	Dabrafenib ± trametinib (MEK inhibitor)	–	NCT01336634	II
	Vemurafenib	–	NCT02314481	II
C-MET	Cabozantinib	–	NCT02132598	II
	Glesatinib (MGCD265)	–	NCT02544633	II
	INC280 + erlotinib	–	NCT01911507	I
	INC280 + gefitinib	–	NCT01610336	Ib/II
VEGFR2/3	Famitinib	–	NCT02356991	II
	Apatinib	Placebo	NCT02332512	III
HER2	Neratinib ± temsirolimus	–	NCT01827267	II
	Afatinib	–	NCT02369484	II
	Trastuzumab emtansine	–	NCT02289833	II
RET	Lenvatinib	–	NCT01877083	II
	Apatinib	–	NCT02540824	II
	Vandetinib	–	NCT01823068	II
	Ponatinib	–	NCT01813734	II
NTRK	Cabozantinib (trial includes RET or ROS1 fusion-positive, increased MET, and AXL NSCLC)	–	NCT01639508	II
	Entrictinib (basket trial for solid tumors, including ROS1 or ALK gene rearrangement)	–	NCT02568267	II

## Author Contributions

First author PB – prepared the main manuscript and table. Corresponding author YB – provided guidance in preparing the manuscript, contributed to literature review and vital modifications, and also contributed to the main manuscript and table. Coauthor DO – prepared part of the manuscript and pathway diagram. Coauthor GG – helped with review, edition, and vital updates to the article. Coauthor GS – helped with editing and review of the manuscript.

## Conflict of Interest Statement

The authors declare that the research was conducted in the absence of any commercial or financial relationships that could be construed as a potential conflict of interest.
